# Reflections on a decade of service as founding Editor-in-Chief of *Conservation Physiology*

**DOI:** 10.1093/conphys/coad103

**Published:** 2024-02-14

**Authors:** Steven J Cooke

**Affiliations:** Fish Ecology and Conservation Physiology Laboratory, Department of Biology and Institute of Environmental and Interdisciplinary Science, Carleton University, 1125 Colonel By Dr., Ottawa, ON, K1S 5B6, Canada

Ten years has come and gone so very quickly. I was a wee bit younger when Craig Franklin and Tony Farrell cornered me at the 2011 Society for Experimental Biology (SEB) Annual Meeting in Glasgow for some very early chats about the potential for a new journal. I left that meeting invigorated and was soon paired with Lindsay Haddon, a previous Managing Editor for the journal *Functional Ecology*, who was recruited to help launch the first new SEB journal in more than a decade. Lindsay and I worked closely with Craig, Tony and then SEB Executive Director Paul Hutchings (the best thinking happened in various Bloomsbury pubs) over the next 1.5 years to develop an ‘academic case’ and a ‘business case’ for a new applied physiology journal. After some refinement, we approached various publishers and came to a co-ownership agreement (50% each) with Oxford University Press (OUP).

What was particularly unique was the fact that this would be the first exclusively Open Access SEB journal. Times were changing such that starting a new subscription-based journal was simply not possible. That was a daunting task; starting a new open access journal in disciplines for which that was out of the norm. We did not rush into it. Rather, with the support of the SEB Board as well as the publisher (OUP), it was decided that there needed to be a period where OA fees were waived. As Editor-in-Chief (EiC), I was worried I would have had to beg for that, but the beauty of SEB is that it is RUN by scientists who understand the realities of other scientists. That investment paid off—you have ‘to spend money to make money’ I am told!

The first paper we ever published was one where we defined the discipline of ‘conservation physiology’ (Cooke *et al.,* 2013), essentially serving as the scope of the journal. Early on, we invited strategic content (e.g. invited reviews or perspective articles on emerging topics) and we continue to do so. For better or worse, our Impact Factor has always been above 2, and at or above that of peer journals (most of which are also between 2 and 3; Note—we are signatories to the Declaration on Research Assessment—or DORA—and are making efforts to identify other mechanisms to assess impact). There are many examples of where the papers we have published contribute to conservation success stories (discussed in Madliger *et al.,* 2016). Our editorial team is always filled with rock stars that span regions, taxa, disciplines and career stages. We recognized that was unique and created opportunities for our editorial team to engage in collaborative papers on topics such as horizon scans (Cooke *et al.,* 2020), research agendas (Cooke *et al.,* 2021) and reflective pieces focused on creating a more inclusive, just and fair community (Cooke *et al.,* 2022*b*).

Given that this is a ‘Voices in Conservation Physiology’ article, I wanted to reflect on some aspects from the last decade. First and foremost, conservation science and research more broadly has evolved such that there is now recognition that not only is this discipline needed (Madliger *et al.,* 2021) but that it can deliver real solutions to benefit biodiversity and people (Madliger *et al.,* 2016; Cooke *et al.,* 2022*a*, 2022b). As a researcher, I have evolved to spend more time thinking about *how* we do science rather than *what* the science has to say. Science done in ways that leaves collateral damage and fails to adequately and respectfully engage stakeholders and rightsholders is not good science (Cooke *et al.,* 2022*a*). The next generation understands this, which gives me much hope. Spending time training the next generation of problem solvers is rewarding; I love nothing more than when a prospective trainee contacts me excited about the notion of working with physiological concepts, tools and knowledge to change the world—when that work is done in a considerate way. In terms of my research programme, I continue to be excited about the opportunity to unravel and unpack the mechanisms underlying conservation problems and to use that knowledge to develop conservation solutions (Cooke *et al.,* 2023). I am by all accounts a ‘fish head’—a fish biologist. But what *Conservation Physiology* has done is open doors such that I think more broadly and consider how every paper I read—regardless of the ecosystem, taxa or geographic scope—applies to my/our work. That is a gift—something I will forever be grateful for and a hallmark of what our journal (and community) stand for.

As I reflect on the last 10 years, I think there are a few things we have done particularly well. First, we pride ourselves on being exceptional communicators—at the level of the journal and the editor. As an author, I can attest that it is all too common to ping a journal/editor about the status of a paper submitted 100+ days ago only to be met with silence. Our goal has always been to be proactive. There will always be papers for which it is challenging to secure referees, and there will always be periods when editors are swamped and cannot devote all their time to the journal. What we strive to do at *Conservation Physiology* is to always respond to queries within a few days and to communicate any challenges we are facing with the authors before they have to reach out to us. Life happens. We feel that communicating to authors that we are doing our best and have not forgotten about them goes a long way. Secondly, we have always been attentive to our community. From organizing conference sessions, to showcasing the work of early career scholars or researchers from under-represented groups and nations, to building science communication expertise using our ‘Conservation Physiology in Action’ series (huge kudos to the lead, Professor Jodie Rummer, our amazing volunteer illustrator, Erin Walsh, and our many early career writers), we have always viewed *Conservation Physiology* as more than just a journal. I also wish to thank the two plant editors (Lauren Sack and then Kevin Hultine) who have handled the bulk of the plant papers.

As EiC, I have adopted a balanced approach to peer review. Being an online-only journal means we do not need to worry about meeting arbitrary page limits each year. Rather, we focus on curating the best possible content to serve our community. Where other journals might say ‘reject’ simply because they need to reduce the number of papers they publish, we often give authors an opportunity to try to address key issues. This would not be possible without thoughtful and thorough referees who provide the authors with lucid guidance to assist them in crafting a paper that is not only publishable, but one that can have real impact. We publish papers that are highly cited, guide our discipline and are the foundation for future work; we also publish papers that may never be cited, but are read and used by practitioners and policy makers, thus supporting real conservation action. Of course, the latter is not reflected in Impact Factor but, frankly, we do not care… putting a value on a conservation decision that benefits biodiversity and people is not easily quantified and we are fine with that!

**Figure 1:**
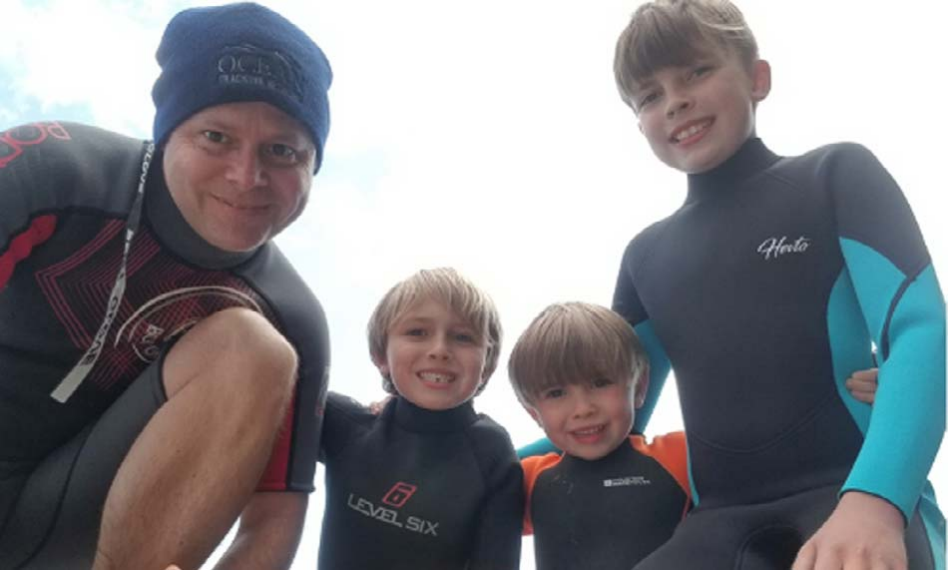
Science is a team sport. I am fortunate to be able to involve my kiddos in research while making memories together. Here, Joshua, Benjamin and Cameron join me for a chilly early-spring snorkelling trip to check the status of black bass parental care on a lake in eastern Ontario. I am grateful to my family for their steadfast love and support.

I can confidently conclude that serving in the EiC role for *Conservation Physiology* has been the single most intellectually stimulating and professionally meaningful role I have had in my career. Helping to define a nascent discipline, build a community of practice and support the next generation of conservation physiology scholars and practitioners makes the role of Editor-in-Chief nothing short of dreamy. As an applied fish ecologist by training, I would have never imagined I would be handling papers about urban racoons with diabetes-like health signatures (Schulte-Hostedde *et al.,* 2018), articles on responses of ants to different thermal conditions (Diamond *et al.,* 2018) or the role of stable isotopes in plant conservation (Snyder *et al.,* 2022). I recall being at the Toronto Zoo at the red panda exhibit and proclaiming to my three children that I was handling a paper on endometrial stem cell collection from that species (Wang *et al.,* 2022). Needless to say, my kids (Figure 1) were underwhelmed, but I still felt cool!

I did want to take a few moments and reflect on the research I have been involved with in the conservation physiology space. I was fortunate to have opportunity to live and breathe this concept while a post-doctoral researcher at the University of British Columbia where we were focused on trying to understand why a particular run of sockeye salmon was migrating to natal spawning grounds early and why those early migrants experienced remarkably high levels of mortality (Hinch *et al.,* 2012). That was my first foray into ‘big science’ and bridging disciplines. Since then, our collaborative work in British Columbia has grown to include further conservation physiology work to address fish passage issues, understand fisheries interactions and to unravel the consequences of climate change on wild salmon (summarized in Cooke *et al.,* 2012). Elsewhere, we have worked on marine and freshwater fishes to generate best practices for catch-and-release recreational fisheries (see Brownscombe *et al.,* 2017). Our fundamental work on understanding the ‘ecology of stress’ has also provided contextual information for our findings (e.g. Lawrence *et al.,* 2017; Birnie-Gauvin *et al.,* 2021). There are a few notable take-home messages from all of these experiences. The first is that mechanisms matter for conservation (Cooke *et al.,* 2023). *Conservation Physiology* is about revealing the mechanisms that underpin conservation problems (e.g. why is a population declining) and thus provides managers with levers they can use to address the root problem. The second is that one cannot do it alone. Conservation problems are complex and the solutions needed to address them will require many minds and much creativity. Working collaboratively is the only way to make meaningful progress—something that certainly rings true in my experiences.

I am forever grateful to the many Associate Editors who have served our journal over the last decade and of course the inner circle of Craig Franklin, Tony Farrell, Lindsay Haddon and various OUP publishers including Jennifer Boyd, Matt Pacey, Nikul Patel and Sarah McKenna. Behind the scenes, there has been OUP marketing staff, various SEB student volunteers operating the Twitter account, production staff and those working in the *Conservation Physiology* editorial office (including Lulu Straeder). Martin Parry was the long-serving SEB Publications Officer and provided much support. Most recently, I wish to acknowledge Bridget O’Boyle as Assistant Editor and Mike Page as SEB Publications Manager for their efforts to help further the journal and ensure SEB values remain front and centre. We are continually grateful to the countless referees who provide free service to our community. Last but not least, thank you to the authors for their high-quality, insightful contributions.

Although I am stepping back, I am not stepping away. I will serve as Emeritus EiC, providing support to the editorial team and helping with strategic planning and other issues that arise. I love writing and have more ideas to tackle with the editorial board. While a journal is never ‘owned’ or ‘controlled’ by one person, I do have an affinity for this one and will continue to be an ardent champion for it. I look forward to submitting my best work to this outlet. Again, a HUGE thanks to everyone for their willingness to support what we believe in here at *Conservation Physiology*. We are better because of you, and I look forward to continuing to be involved with this dynamic, supportive and innovative community.
